# Genome-wide identification and expression analysis of the *TCP* transcription factor family and its response to abiotic stress in rapeseed (*Brassica napus* L.)

**DOI:** 10.1007/s13205-025-04273-x

**Published:** 2025-04-07

**Authors:** Xinrui Xu, Xin Zhang, Yu Fan, Hui Zhou, Xiaobin Pu

**Affiliations:** 1 Crop Research Institute of Sichuan Academy of Agricultural Sciences/Environmentally Friendly Crop Germplasm Innovation and Genetic Improvement Key Laboratory of Sichuan Province, Chengdu, 610066 China; 2 College of Food and Biological Engineering, Chengdu University, Chengdu, 610106 China; 3 Sichuan Province Seed Station, Chengdu, 610041 China

**Keywords:** *Brassica napus*, *TCP* gene family, Evolutionary analysis, Expression profile, Abiotic stress

## Abstract

The study used 80 *BnTCP* genes (*Brassica napus TCP* genes) in rapeseed, which were identified and designated with nomenclature based on their chromosomal locations. A systematic analysis encompassed the evolutionary relationships, classifications, gene structures, motif compositions, chromosome localization, and gene replication events within these *BnTCP* genes. These 80 BnTCP proteins were categorized into three subfamilies, with the PCF subfamily showing significant expansion during evolution. Segmental duplications were identified as a major driver of TCP family amplification. To comprehensively assess the evolutionary relationships of the TCP family across diverse plant species, nine comparative genomic maps were constructed, elucidating homologous genes between *B. napus* and representative monocotyledonous and dicotyledonous plants. In the final phase of the study, the gene expression response characteristics of 15 selected *BnTCP* genes across various biological processes and stress responses were examined. Noteworthy candidates, including *BnTCP28*, *BnTCP30*, and *BnTCP76*, were identified as potentially crucial in tissue development and environmental stress responses.

**Supplementary Information:**

The online version contains supplementary material available at 10.1007/s13205-025-04273-x.

## Introduction

The *TCP* gene family, first identified by Cubas et al. (1999a, b), is a novel transcription factor family, characterized by a limited number of members. The name *TCP* is derived from the initials of three genes: *TB1* (*TEOSINTE BRANCHED 1*) in maize, *CYC* (*CYCLOIDEA*) in snapdragon, and *PCF1* (*PROLIFERATING CELL FACTORS 1*) and *PCF2* (*PROLIFERATING CELL FACTORS 2*) in rice (Doebley et al. 1995; Luo et al. 1996). The *TCP* gene family exhibits a highly conserved domain, representing a common feature among its members (Kim et al. 2014). The distinctive domains within TCP proteins are categorized into two groups: Class I and Class II. The primary disparity between Class I and Class II lies in the absence of four amino acids in the TCP domain of Class I compared to Class II (Cubas et al. 1999a, b). Class II proteins are further subdivided into the CINCINNATA (CIN) and CYCLOIDEA (CYC) proteins (Navaud et al. 2007). In addition, the CYC/TB1 protein is found only in angiosperms, while the CIN protein is found in mosses, which shows that the CIN grade is older than the CYC/TB1 grade (Navaud et al. 2007).

Various experimental methods have validated that *TCP* transcription factors regulate target gene expression by recognizing and binding regions rich in GC sequences within gene promoters (Danisman et al. 2012). Notably, different *TCP* family members exhibit preferences for distinct binding sequences. Class I proteins predominantly bind to GGNCCCAC sequences (Kosugi et al. 2002), while class II proteins prefer GTGGNCCC sequences (Viola et al. 2011). Advances in genome sequencing have facilitated the identification of *TCP* gene family members in numerous plant species, including 29 *TCP* genes in *Zea mays* (Chai et al. 2017), 72 in *Arabidopsis thaliana* (Yao et al. 2007), 52 in *Malus domestica* (Xu et al. 2014), 61 in *Nicotiana tabacum* (Chen et al. 2016), 20 in *Sorghum bicolor* (Francis et al. 2016), 41 in *Panicum virgatum* (Zheng et al. 2019), 27 in *Cucumis sativus* (Wen et al. 2020), 20 in *Broussonetia papyrifera* (Zhao et al. 2020), 35 in *Melastoma candidum* (Li et al. 2022a, b), 14 in *Cymbidium goeringii* (Liu et al. 2022), 23 in *Chrysanthemum nankingense* (Tian et al. 2022), 30 in *Solanum melongena* (Li et al. 2022a, b), 39 in *Chrysanthemum lavandulifolium* (Wu et al. 2023), and 18 in *Rosa chinensis* (Zou et al. 2023). The CIN clade, is ubiquitously present in all identified species, while the CYC clade is exclusive to monocotyledonous plants (Damerval et al. 2022). The evolutionary trajectory of the *TCP* factor highlights the absence of the CYC/TB1 subclass in lower plants, with an independent evolution observed in dicotyledonous and monocotyledonous plants (Zhou et al. 2022). Navaud et al.'s investigation suggested that the *TCP* gene family may have originated from aquatic algae, with lower plants exhibiting a lower abundance of *TCP* genes than higher plants (Navaud et al. 2007).

*TCP* transcription factors play crucial roles in various plant processes, including flower asymmetry, seed germination, leaf development, and defense responses (Danisman et al. 2016; Li et al. 2015). Notably, *TCP* genes play a pivotal role in leaf development. For instance, in *Arabidopsis*, mutations in the class I genes *AtTCP7*, *AtTCP8*, *AtTCP22*, and *AtTCP23* lead to a reduced number of rosette leaves in the mutant. The genes *CYCA1;1* and *CYC 2;3*, which are involved in cell cycle regulation, are upregulated in multiple mutants, leading to enlarged leaves (Aguilar-Martínez et al. 2013). These findings underscore the role of class I genes in regulating leaf development through the control of cell proliferation. The *AtTCP11-EAR* mutant in *Arabidopsis* displays a leaf curl phenotype, indicating that class I genes also influence apical dominance (Viola et al. 2011). In contrast, class II genes primarily regulate leaf development by inhibiting cell proliferation and differentiation. *CIN* subfamily genes modulate leaf shape by restraining the proliferation of leaf margin cells. Multiple mutants of *CIN* subfamily genes lacking *TCP* gene function exhibit overexpression of leaf margin genes, causing continued cell division and increased leaf area, ultimately resulting in wrinkled and curled leaves. Analysis of *CIN* gene expression patterns revealed that these genes further regulate leaf shape and area by controlling cell differentiation (Efroni et al. 2008). The *LA* in tomato, identified as the homologous counterpart of *AtTCP4*, was investigated by Ori et al. (2007), who revealed that the *la* mutation led to the transformation of compound leaves into single leaves. *TCP* genes play pivotal roles not only in leaf development but also in the intricate regulation of plant reproductive structures. For instance, in snapdragon, *CYCLOIDEA*, a CYC subfamily member, modulates floral symmetry (Crawford et al. 2004). Similarly, the CYC orchestrates petal development and floral symmetry in pea, similar to the mechanisms observed in *Antirrhinum majus*, primarily involving the orchestration of floral dorsal and ventral identity (Feng et al. 2006). In addition, specific class I genes in *Arabidopsis*, such as *AtTCP14* and *AtTCP15*, have been shown to contribute to the formation of floral organs (Oshodi et al. 1999; Abugoch James 2009). In *Arabidopsis*, the *AtTCP16* gene, which belongs to the class I *TCP* gene family, plays a crucial role in early pollen development (Takeda et al. 2006). This pattern extends to other species, such as *GhcyC2* in *Gerbera hybrida* (Broholm et al. 2008) and *HaCYC2c* in *Helianthus annuus* (Fambrini et al. 2011), where involvement in regulating flower development has been established. The left and right symmetrical parts of the flowers along the lemma inner axis in rice are indirectly determined by the *RETARDED PALEA1* (*REP1*) gene of the *CYCLOIDEA* (*CYC*)-like homolog, which regulates the identity and development of the lemma. The *REP1* gene is expressed only in the palea primordia during early flower development but is radially dispersed in the vascular bundles of stamens, lemmas, and paleae during later flower development. In the *rep1* mutant, the development of the lemma is significantly delayed (Yuan et al. 2009).

Furthermore, members of the *TCP* gene family are implicated in the response to a spectrum of biotic and abiotic stresses. For instance, the overexpression of the class II gene *OsTCP21* in rice has been shown to heighten sensitivity to low temperatures, suggesting a negative regulatory role in cold resistance (Wang et al. 2014). In rice, the gene *PCF2* affects salt tolerance by positively modulating the expression of the Na + /H + antiporter gene *OsNHX1* (Almeida et al. 2017). In addition, *PCF5* and *PCF6* are linked to drought, salt, and cold stress tolerance, respectively. In maize, the overexpression of *ZmTCP42* has been demonstrated to enhance drought tolerance in plants (Ding et al. 2019). Exposure to abscisic acid (ABA) treatment results in the significant upregulation of seven *DoTCP* genes in *Dendrobium orchids*, indicating a potentially significant role for the *TCP* gene family in hormone signaling (Li et al. 2023). Notably, *PeTCP10* is induced by salt stress and is integral to the regulatory response to salt stress in *Arabidopsis*. Salt stress represents a considerable impediment to plant development, and *PeTCP10* is prominently expressed in the mature leaves, roots, and stems of *Phyllostachys pubescens* under normal conditions, with expression further induced under salt stress (Xu et al. 2021). Moreover, the upregulation of *AtTCP3*, a member of the CIN branch, has been observed to influence the expression of *CYP83B1*/*SUR2*, thereby repressing indole-3-acetic acid (IAA) synthesis in *Arabidopsis* seedlings. *AtTCP3* also modulates the distribution of PIN proteins, which are integral to IAA transport, thereby mitigating the plant’s sensitivity to IAA (Bak et al. 2001). In a similar vein, *AtTCP3*, in concert with CIN genes in *Antirrhinum majus*, activates the expression of the IAA inhibitory gene *IAA3*/*SHY2* (Li et al. 2013; Koyama et al. 2010a, b). These stress responses are peculiar to terrestrial plants, underscoring the importance of comprehensive genome-wide analyses of the *TCP* family for understanding the adaptive and evolutionary dynamics of various plant species within their respective environments.

The emergence of rapeseed as a cultivated crop is a relatively recent development within the history of agriculture, having been domesticated for millennia without a known wild progenitor (Das et al. 2014). As a pivotal crop for the production of edible oil, vegetable products, biofuels, and animal feed, rapeseed also holds ornamental value due to its array of petal colors (Mason et al. 2016). Globally, rapeseed stands out as a crucial oilseed crop, with its production volume second only to soybean. Cultivated across approximately 35 million hectares in regions including China, Europe, Canada, and Australia, it yields an impressive annual output of 70 million tons of oilseeds (Hu et al. 2021). The importance of rapeseed transcends its sheer production volume, as it contributes to the circular economy through its abundant supplies of vegetable oil and protein-rich meal (Lu et al. 2019). The origins of rapeseed can be traced back to Europe, where it originated from spontaneous hybridization events between cabbage (2n = 20, AA) and kale (2n = 18, CC) (Zou et al. 2018). This botanical lineage highlights the genetic intricacy and evolutionary trajectory of rapeseed, elucidating its distinctive attributes and adaptability. The cultivation and utilization of rapeseed are integral to global agricultural practices, playing a crucial role in meeting the demand for vegetable oil and protein resources. The extensive expansion of rapeseed agriculture across diverse geographical areas underscores its adaptability and economic importance on a worldwide scale. In the context of *B. napus*, the selection of seed types has led to the accelerated loss of glucosinolate genes while concurrently promoting the expansion of genes associated with oil biosynthesis. These evolutionary processes offer valuable insights into the dynamics of allopolyploid evolution and its interplay with crop domestication and improvement (Chalhoub et al. 2014). To date, comprehensive investigations into rapeseed have been limited. This study aims to fill this void by conducting a systematic analysis of the *BnTCP* gene family in rapeseed. We have meticulously identified 80 *TCP* genes in *B. napus* and explored their gene structure, motif composition, duplication events, chromosomal distribution, and phylogenetic relationships. Through these analyses, we seek to elucidate the evolutionary history and functional diversity of the *TCP* gene family in rapeseed, as well as to infer the potential roles of specific *TCP* genes in various biological processes. Furthermore, this study investigates the expression profiles of *BnTCP* genes under a range of stress conditions and hormone treatments. This in-depth analysis is designed to uncover the nuanced responses of these genes to different environmental cues. The findings from these experiments provide valuable insights into the functional identification and evolutionary relationships of *BnTCP* genes in rapeseed. This comprehensive approach not only enhances our understanding of the molecular mechanisms within the *BnTCP* family but also lays the groundwork for future research in this domain.

## Materials and methods

### Gene identification

We retrieved the latest high-quality reference genome of *B. napus* from the National Center for Biotechnology Information (NCBI) GenBank website (accession number GCF_020379485.1) (Schoch et al. 2020). To identify candidate TCP proteins in rapeseed, we initiated our analysis by employing all TCP proteins of *Arabidopsis* (72 AtTCP) (https://doi.org/10.1111/j.1744–7909.2007.00509.x) as queries in a BLASTp search against the rapeseed genome (Altschul et al. 1997). The candidate genes were selected based on stringent criteria of a score ≥ 100 and an *e* value ≤ e − 10. Subsequently, we utilized the Hidden Markov Model (HMM) file of the TCP domain (PF03634) obtained from the PFAM protein family database (http://pfam.xfam.org/). Using the HMMER3.3 online software with a decision value of 0.01 (https://www.ebi.ac.uk/Tools/hmmer/) (Finn et al. 2011), we identified TCP protein sequences in *B. napus*. Conserved motifs within the TCP proteins in rapeseed were identified through PFAM and SMART via thread sequencing (http://smart.embl-heidelberg.de/) (Bateman et al. 2000; Letunic et al. 2018). This meticulous process resulted in the identification of a total of 80 BnTCP proteins in rapeseed. To ensure the accuracy of our findings, we subjected these identified BnTCP proteins to reverification in the NCBI protein database using the BLASTp program (https://blast.ncbi.nlm.nih.gov/Blast.cgi ? PROGRAM = BLASTP& PAGETYPE = BlastSearch&LINKLOC = blast home). Finally, we used the ExPASy online program to elucidate the basic features of the *TCP* gene in *B. napus*, including sequence length, protein molecular weight, isoelectric points, and subcellular localization (http://web.ExPASy.org/protparam/).

### *TCP* gene structures and conserved motif analysis

The exon‒intron substructure of the *BnTCP* genes was analyzed using the Gene Structure Display Server (GSDS; http://gsds.cbi.pku.edu.cn) online software (Guo et al. 2007). Subsequently, the conserved motifs and structural differences in the TCP proteins were investigated using the MEME online program (http://meme.nbcr.net/meme/intro. HTML) (Bailey et al. 2009). The optimization parameters for identifying conserved motifs were set to a maximum of 10. In addition, cis-acting elements in the upstream 2000 bp promoter region of 80 *TCP* genes were predicted using PlantCARE online software (http://bioinformatics.psb.ugent.be/webtools/plantcare/html/?tdsourcetag=spcqqaiomsg) (Lescot et al. 2002). These analytical approaches provide comprehensive insights into the genetic architecture of *BnTCP* genes, revealing both their structural features and conserved motifs.

### Chromosomal distribution and gene duplication

Initially, all *BnTCP* genes were assigned chromosomal details based on their physical location in the annotation file. Subsequently, Circos software was used to analyze the chromosomal location information of these *BnTCP* genes (Krzywinski et al. 2009). Gene replication events were investigated using the Multiple Collinear Scanning Toolkits (MCScanX) with default parameters (Finn et al. 2011; Wang et al. 2012). To assess the homology of *TCP* genes between *B. napus* and nine other plants (*O. sativa*, *Z. mays*, *S. bicolor*, *A. thaliana*, *C. sativus*, *V. vinifera*, *B. rapa*, *B. oleracea*, and *B. juncea*), a dual synteny plotter project was constructed using TBtools software (v2.038) (Chen et al. 2020).

### Phylogenetic analysis and classification of the *BnTCP* family

According to the classification of AtbZIP proteins, the 80 TCP proteins in *B. napus* were categorized into three main subfamilies. For the construction of neighbor-joining (NJ) trees, the Jukes–Cantor model was employed using MEGA 7.0 (https://www.megasoftware.net/). The bootstrap value of the phylogenetic tree was set to 1000, and the tree was generated with Geneious R11 using the BLOSUM62 cost matrix. In addition, a multispecies phylogenetic tree was created, incorporating TCP protein sequences from rapeseed and *A. thaliana* sourced from the UniProt website (https://www.uniprot.org/). This study utilized robust phylogenetic analysis methods, including NJ trees with high bootstrap support, to elucidate the evolutionary relationships within the identified subfamilies of TCP proteins in *B. napus*.

### Plant materials, growth conditions, and abiotic stresses in *B. napus*

This investigation utilized *B. napus* cv. *Tianfuyou*, a well-established traditional variety indigenous to Sichuan Province in Southwest China. Since 2022, the cultivation of 'Tianfuyou' has been carried out in greenhouse facilities at the Sichuan Academy of Agricultural Sciences Farm. The seedlings were initially sown in seedling trays within the greenhouse. Subsequently, the rapeseed plants were transplanted into pots containing a substrate mixture of equal parts soil and vermiculite and were then placed in a controlled growth chamber. The environmental conditions within the growth chamber were carefully managed to maintain a diurnal cycle, with 16 h of daylight at 25 °C and 8 h of darkness at 20 °C, while the relative humidity was kept at 75%. A nutrient solution consisting of 10 g of urea, 6 g of potassium dihydrogen phosphate, 2 g of calcium sulfate, and 1 g of magnesium sulfate dissolved in 20 L of water was applied monthly. Each application involved spraying 300 mL of the solution onto the potted plants. In addition, standard irrigation with pure water was applied approximately every 5 days. For a comprehensive analysis, samples were collected from the roots, stems, leaves, flowers, seeds, and seed coats during the early ripening stage of the rapeseed plants. For seed-specific analyses, particular attention was given to five key developmental stages: 7 day post-anthesis (early filling stage), 14 days (mid-filling stage), 21 days (early ripening stage), 28 days (mid-ripening stage), and 35 days (full ripening stage) following anthesis.

Each organ sample was meticulously obtained from five individual plants, which were cultivated under uniform growth conditions to ensure the reliability and consistency of the data. To preserve the physiological state of the samples, a rapid collection technique was employed, in which the samples were immersed in liquid nitrogen that had been precooled to maintain their molecular integrity. Subsequently, the collected samples were stored at −80 °C for subsequent analysis. Total RNA extraction was performed promptly on these samples to facilitate subsequent quantitative reverse transcription polymerase chain reaction (qRT-PCR) analysis. Each sample was processed with at least three technical replicates to ensure the robustness of the gene expression data and to minimize experimental variability. In addition to organ-specific analysis, we investigated the expression patterns of *BnTCP* genes in rapeseed seedlings subjected to diverse abiotic stresses and hormone treatments. The rapeseed seeds were initially sown in seedling trays, and after a germination period of 28 days, the seedling trays were treated with 50 mL of solution, and the seedling roots were effectively submerged. The four distinct abiotic treatments administered to *B. napus* plant seedlings included ultraviolet radiation salt (5% NaCl), drought (10% PEG6000), high temperature (40 °C), and cold (4 °C). In addition, four hormone treatments involving gibberellin (GA3, 100 µM), auxin (indole-3-acetic acid, IAA, 100 µM), and abscisic acid (50 µM) were applied. To ensure comprehensive analysis and statistical robustness, each stress treatment was conducted in five parallel sets. The expression patterns of genes in the leaves, roots, and stems of rapeseed plants were examined at distinct timepoints (0, 1, 4, and 12 h) following stress induction.

### Total RNA extraction, cDNA reverse transcription, and qRT‒PCR analysis

Extraction of total RNA from fresh tissue samples was conducted using the TianGen RNA Easy Fast Plant Tissue Kit (DP452), a kit renowned for its efficiency and dependability in plant RNA extraction. Following RNA extraction, reverse transcription was executed with the TianGen FastKing One-Step RT-qPCR Kit (SYBR, FP313–01), which is designed for use with SYBR Green chemistry and features an optimized one-step protocol. To facilitate precise and gene-specific quantification, primers for qRT-PCR were meticulously designed for all genes using Primer 5.0 software (Table S1). The use of Actin as the internal control provides a stable reference for normalization across samples. The standard expression for SYBR Premix ExTaqII (Takara Bio) was replicated on a CFX96 real-time system (Bio-Rad), and the entire process was repeated three times. The 20 μL qRT‒PCR system included 1 μL cDNA (100 ng/μL-1), 10 μL SYBR Green Real-time PCR Master Mix (Takara Bio), 0.5 μL each of forward and reverse primers, and 8 μL RNase-free water. This well-balanced composition ensures optimal amplification conditions. To validate the reliability of the quantitative primers, melting curve analysis was performed, confirming their specificity and eliminating the possibility of nonspecific amplification or primer–dimer formation. The expression of these *TCP* genes was analyzed using the 2^− (ΔΔCt)^ method (Livak et al. 2001).

### Statistical analysis

The least significant difference test (LSD) was employed, with a significance level set at 0.05, using JMP 6.0 software for statistical analysis. Origin 2016 from OriginLab Corporation, Northampton, Massachusetts, USA, was utilized for drawing histograms. To evaluate the correlation among *BnTCP* genes, the Pearson correlation coefficient was determined using SigmaPlot 12.0 software. The correlation coefficient was defined as *P* < 0.05.

## Results

### Identification of *TCP *genes in *Brassica napus*

Two BLAST methods were employed to systematically identify *TCP* genes in the genome of *Brassica napus* (Table S2). The identified genes were subsequently named *BnTCP1* to *BnTCP80* based on their specific locations in the rapeseed chromosomes. A comprehensive analysis was conducted to elucidate their essential characteristics, including gene coding sequence (CDS), protein molecular weight (MW), isoelectric point (pI), and subcellular localization.

Within the set of 80 BnTCP proteins, BnTCP20 is distinguished as the smallest, comprising 213 amino acids, whereas BnTCP26 is the largest, consisting of 479 amino acids (Table S2). The molecular weights of these proteins ranged from 22.98 kDa (BnTCP78) to 51.68 kDa (BnTCP26). The pI values ranged from 5.4 (BnTCP46) to 10.38 (BnTCP10), with an average of 7.57. Notably, all BnTCP proteins harbored the TCP domain. Subsequent to subcellular localization predictions, 76 BnTCP proteins were identified in the nucleus, one (BnTCP15) in the chloroplast and nucleus, two (BnTCP23 and BnTCP61) in peroxisomes, and one (BnTCP60) in the endoplasmic reticulum and plasma membrane (Table S2). The ratio of *BnTCP* genes to total genes in the *B. napus* genome was approximately 0.06%, which is lower than that in *Arabidopsis* (0.09%) (Yao et al. 2007), tomato (0.09%) (Parapunova et al. 2014), and maize (0.12%) (Chai et al. 2017) but higher than that in sorghum (0.05%) (Zheng et al. 2019). This information provides valuable insights into the evolutionary patterns and diversification of the *TCP* gene family across various plant species.

### Phylogenetic analysis and classification of *BnTCP* genes

**Fig. 1:**
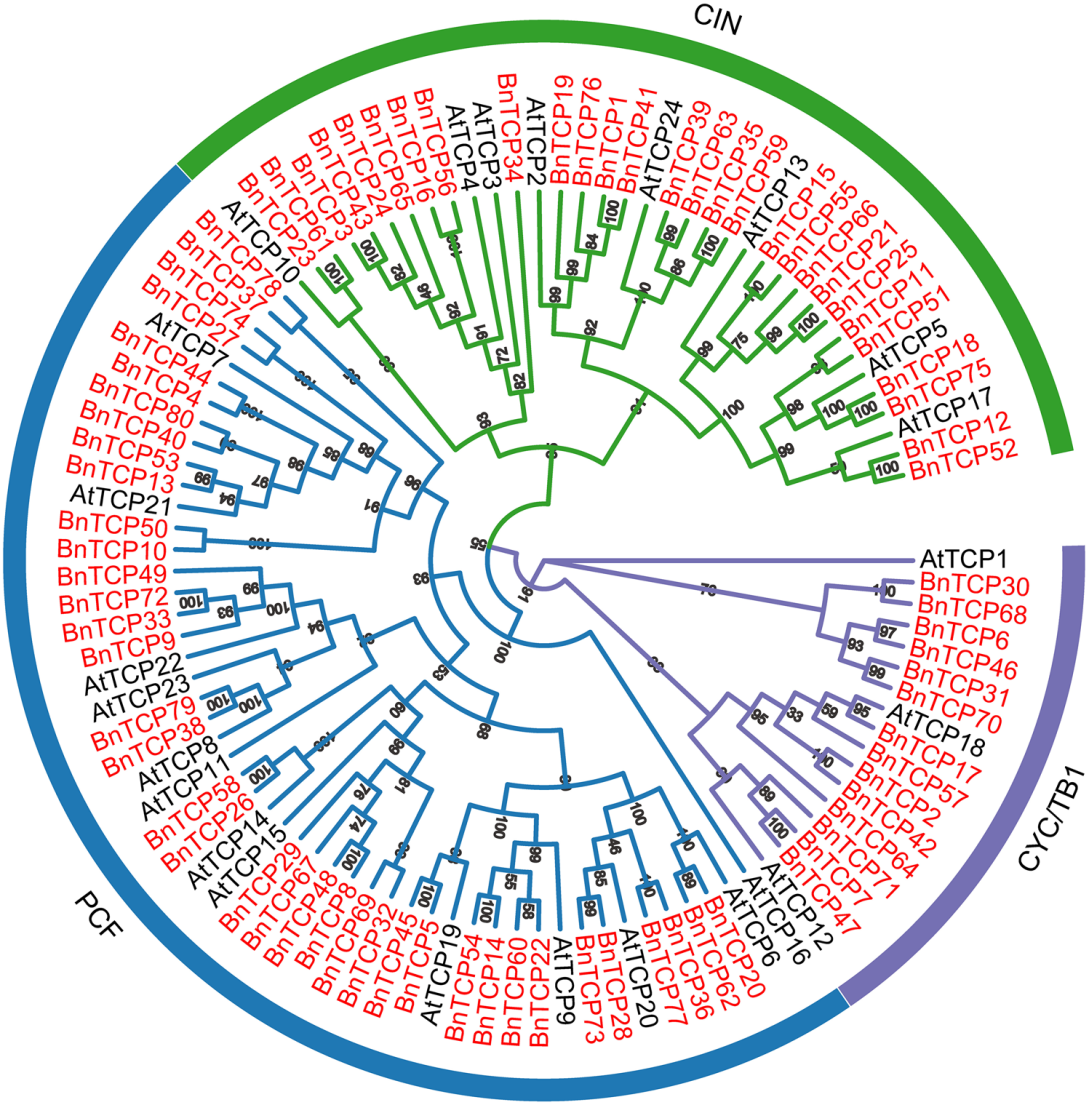
Unrooted phylogenetic tree showing relationships among *TCP* genes of *Brassica napus* and *Arabidopsis*. The phylogenetic tree was derived using the neighbor-joining method in MEGA7.0. The tree shows the three phylogenetic subfamilies. TCP proteins from *Arabidopsis* is denoted by the prefix ‘At’

To delineate the subfamily classification of TCP proteins in *B. napus*, we constructed phylogenetic trees encompassing 80 BnTCP proteins from *B. napus* and 24 AtTCP proteins from *A. thaliana* using the neighbor-joining method (Fig. 1, Table S2). According to the classification method and topological structure proposed by Yao et al. (2007), the 80 TCP proteins within the phylogenetic tree were categorized into three primary branches. This finding aligns seamlessly with the established classification of the TCP subfamily in *Brassica* plants. These data indicate that these proteins were not lost in *B. napus* during the evolutionary process. These subfamilies of TCP proteins (Class I) exhibit widespread presence across diverse angiosperms, suggesting their fundamental roles in plant development and evolution. This finding aligns with recent findings reported across diverse plant species, such as *A. thaliana* (Yao et al. 2007), *N. tabacum* (Chen et al. 2016), *S. bicolor* (Francis et al. 2016), and *P. virgatum* (Zhao et al. 2020). The conserved nature of these proteins across different plant lineages highlights their essential contributions to the intricate processes of plant biology. Among the three subfamilies, PCFs had the highest membership, with 38 BnTCP proteins, while CYC/TB1, with only 14 members, represented the subfamily with the fewest members (Fig. 1, Tables S2 and S3). Notably, CYC/TB1 and CIN, both of which belong to Class II, collectively comprise 42 TCP protein members, providing insights into the diversity within this class. Examining the relationships between BnTCP proteins and AtTCP proteins revealed some proteins in *B. napus* that form tight clusters with AtTCP proteins (guiding support ≥ 60). This suggests the possibility of orthologous relationships between these proteins in *B. napus* and *Arabidopsis*, implying similar physiological functions.

### Conserved motif, gene structure, and cis-acting element analysis of *BnTCP* genes

**Fig. 2:**
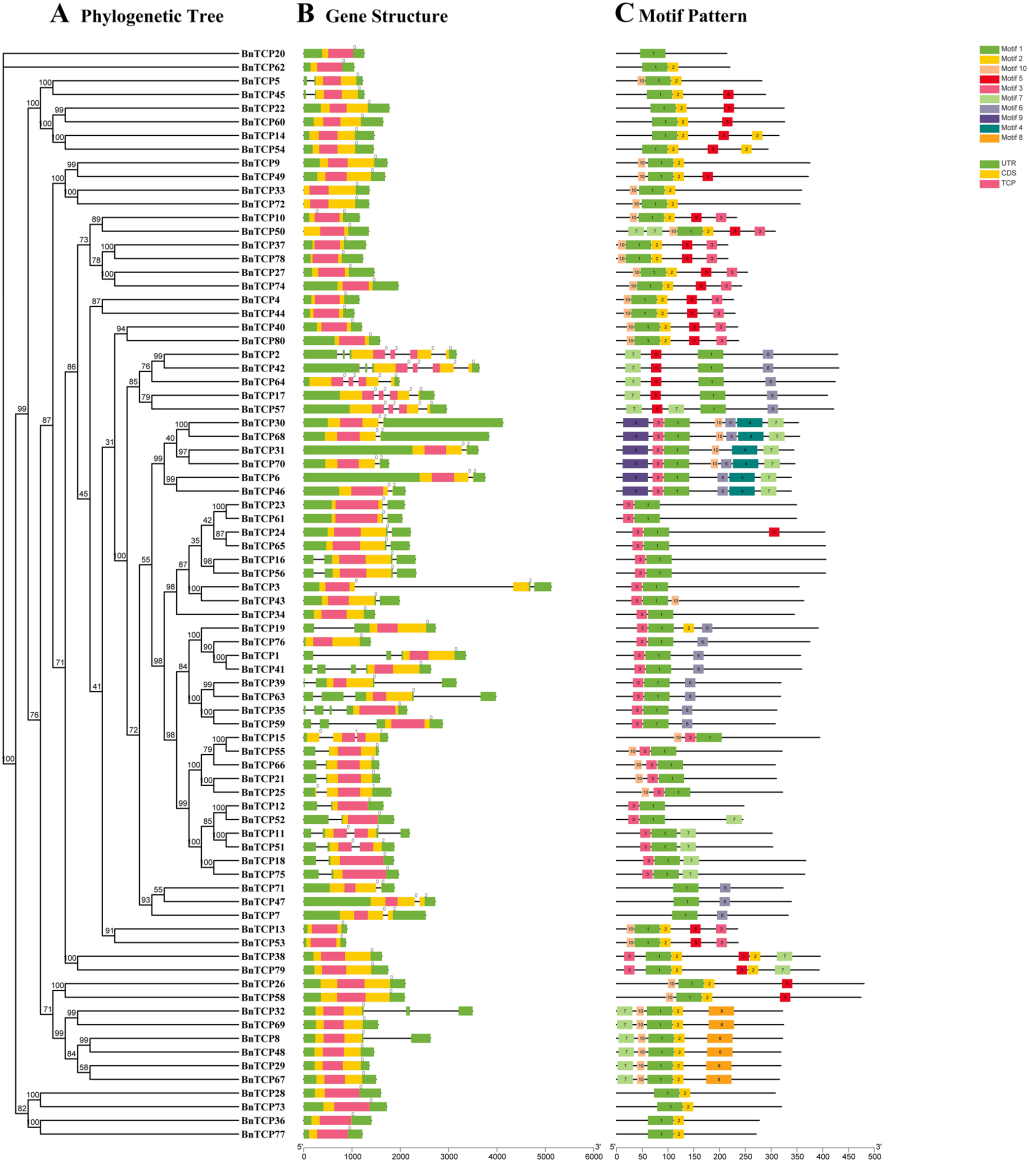
Phylogenetic relationships, gene structure analysis, and motif distributions of *B. napus TCP* genes. **A** Phylogenetic tree was constructed using the neighbor-joining method with 1000 replicates for each node. **B** Exons and introns are indicated by yellow rectangles and grey lines, respectively. **C** Amino acid motifs in the BnTCP proteins (1–10) are represented by colored boxes. The black lines indicate relative protein lengths

Through a comprehensive examination of the DNA structures within the *B. napus* genome, we conducted an in-depth analysis of the number and arrangement characteristics of exons and introns among BnTCP members (Fig. 2, Table S2). Our analysis revealed that the 80 *BnTCP* genes display a spectrum of exon counts, ranging from one to six. Interestingly, 35 (43.75%) of the BnTCP proteins contain a single exon and are represented within two separate subfamilies, the PCF and CIN subfamilies. In contrast, the remaining genes are characterized by the presence of two or more exons. Notably, members of the CYC/TB1 subfamily, such as *BnTCP2* and *BnTCP42*, exhibit the greatest number of exons (6). Notably, CYC/TB1 subfamily members share analogous complex genetic structures, typically containing 2, 4, or 6 exons. Subfamily PCFs predominantly encompass one or two exons, with the exception of specific genes, such as *BnTCP32*. Conversely, the CIN subfamily demonstrated the highest diversity in exon types, featuring four distinct exon types. Despite potential inconsistencies in exon and intron distributions, members within the same subfamily generally exhibit structural similarities.

The MEME online program identified ten conserved motifs in these BnTCP proteins, and the characteristic amino acid regions of the BnTCP members were systematically organized (Fig. 2, Table S3). Motif 1 is ubiquitously distributed across all TCP proteins, and the positions of these motifs are typically closely aligned in most members. In general, BnTCP proteins within the same subfamily share similar motif arrangements. For instance, subfamily CYC/TB1 is characterized by motifs 7 and 6, subfamily PCF features motifs 10 and 2, and subfamily CIN includes motifs 1 and 3. In addition, specific motifs are exclusive to particular subfamilies; for instance, motif 9 is unique to subfamily CYC/TB1, and motif 8 is specific to subfamily PCF. Further analysis revealed the consistent positioning of certain motifs within the structures of BnTCP proteins, such as motif 3 in the CIN subfamily, which mostly appears at the beginning of this pattern. Motifs 7 and 9 mainly occupied the starting position of the CYC/TB1 subfamily, while motif 10 appeared mostly at the beginning of the PCF subfamily. Notably, in the CYC/TB1 subfamily, motif 7 is always present at the end of this pattern. The repeated arrangement of these motifs in the same subfamily emphasizes the similarity of motif structure, which means that the protein structure among members of the same subfamily is conserved. This observation supports the robustness of phylogenetic tree classification.

To unravel the complex physiological processes governed by these genes, we conducted a detailed examination of the cis-acting elements within the promoter regions (upstream 2000 bp) of 80 *BnTCP* genes. Comprehensive identification revealed 106 cis-regulatory elements (Table S4), corresponding to 57 distinct physiological functions. These cis-regulatory elements can be categorized into eight groups: site-binding-related, development-related, environmental stress-related, hormone-responsive, light-responsive, promoter-related, wound-responsive, and miscellaneous. The predominant category among the identified promoter elements of the *BnTCP* genes was promoter-related elements, which encompassed five cis-regulatory factors. Notably, five promoter-related elements (CAAT-box, TATA-box, A-box, Box II-like sequence, and 3-AF3 binding site) were universally present in all *BnTCP* genes. Within the 80 *BnTCP* genes of *B. napus*, twelve hormone-responsive elements were identified, covering a spectrum of plant hormones, including abscisic acid-responsive (ABRE, AAGAA-motif), auxin-responsive (AuxRR-core, AuxRE, and TGA-box), gibberellin-responsive (P-box, GARE-motif, and TATC-box), salicylic acid-responsive (TCA-element), and jasmonic acid-responsive (TGACG-motif, CGTCA-motif) elements. Furthermore, cis-regulatory elements associated with drought, low temperature, salt stress, anaerobic conditions, and other defense and stress responses were identified among the *BnTCP* genes. This comprehensive analysis sheds light on the intricate regulatory networks governing the diverse physiological functions of these genes. Approximately 67.50% of the *BnTCP* genes exhibited abscisic acid (ABA)-responsive and methyl jasmonate (MeJA)-responsive elements, while approximately 65.00% of the *TCP* genes harbored gibberellin-responsive elements. A comprehensive analysis revealed twelve cis-acting elements orchestrating regulatory processes in diverse tissues (endosperm, meristem, leaf, root, and seed) during different developmental stages of *B. napus*. Consequently, *BnTCP* genes not only actively contribute to the developmental processes of multiple tissues but also demonstrate responsiveness to various abiotic stresses and wounds.

### Chromosomal spread and gene duplication of *BnTCP* genes

**Fig. 3:**
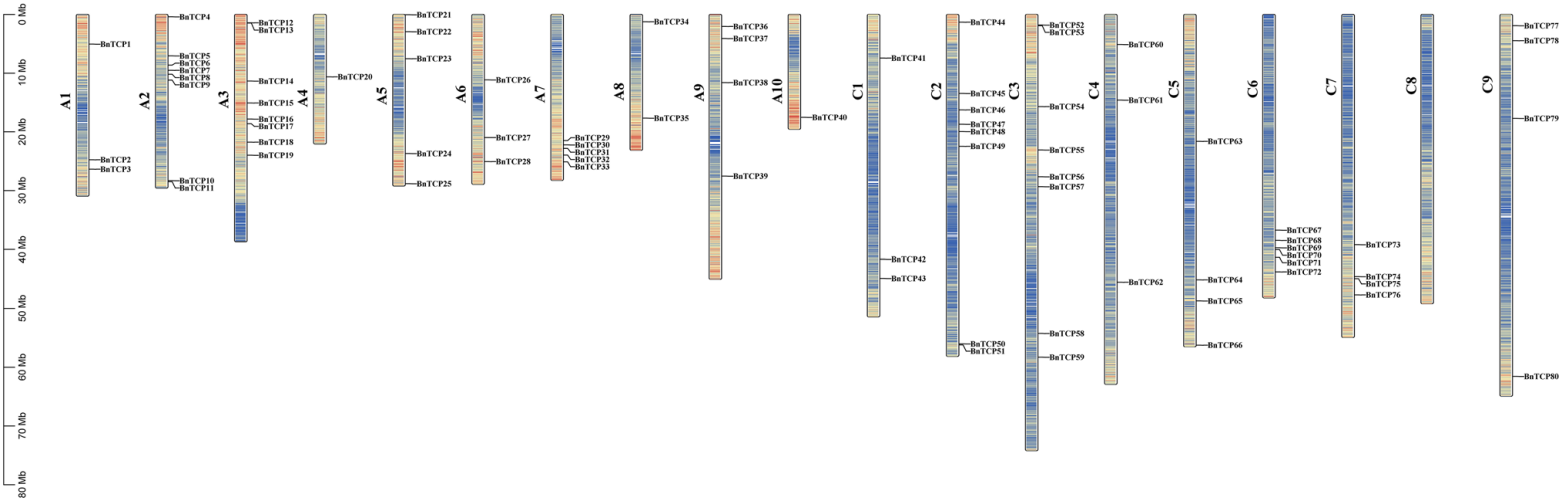
Schematic representation of the chromosomal distribution of the *B. napus TCP* genes. Vertical bars represent the chromosomes of *B. napus*. The chromosome number is indicated to the left of each chromosome. The scale on the left represents chromosome length

A total of 80 *BnTCP* genes exhibited an uneven distribution across chromosomes A1–A10 and C1–C9 (Fig. 3, Table S2). Each *BnTCP* gene is mapped to a distinct physical location on various chromosomes in *B. napus*. Notably, chromosomes A3 and C3 had the greatest number of *BnTCP* members, each hosting eight *BnTCP* genes, accounting for approximately 10.00% of the total, followed closely by A2 and C2, with 7 genes each (approximately 8.75%). In contrast, C8 lacked any *BnTCP* genes. Further chromosomal breakdown revealed that A1, A4, A5, A6, A7, A8, A9, and A10 contained 3 (~ 3.75%), 1 (~ 1.25%), 5 (~ 6.25%), 3 (~ 3.75%), 5 (~ 6.25%), 2 (~ 2.50%), 4 (~ 5.00%), and 1 (~ 1.25%) *BnTCP* genes, respectively. In addition, chromosomes C1, C4, C5, C6, C7, and C9 harbor 3 (~ 3.75%), 3 (~ 3.75%), 4 (~ 5.00%), 6 (~ 7.50%), 4 (~ 5.00%), and 4 (~ 5.00%) *BnTCP* genes, respectively.

**Fig. 4:**
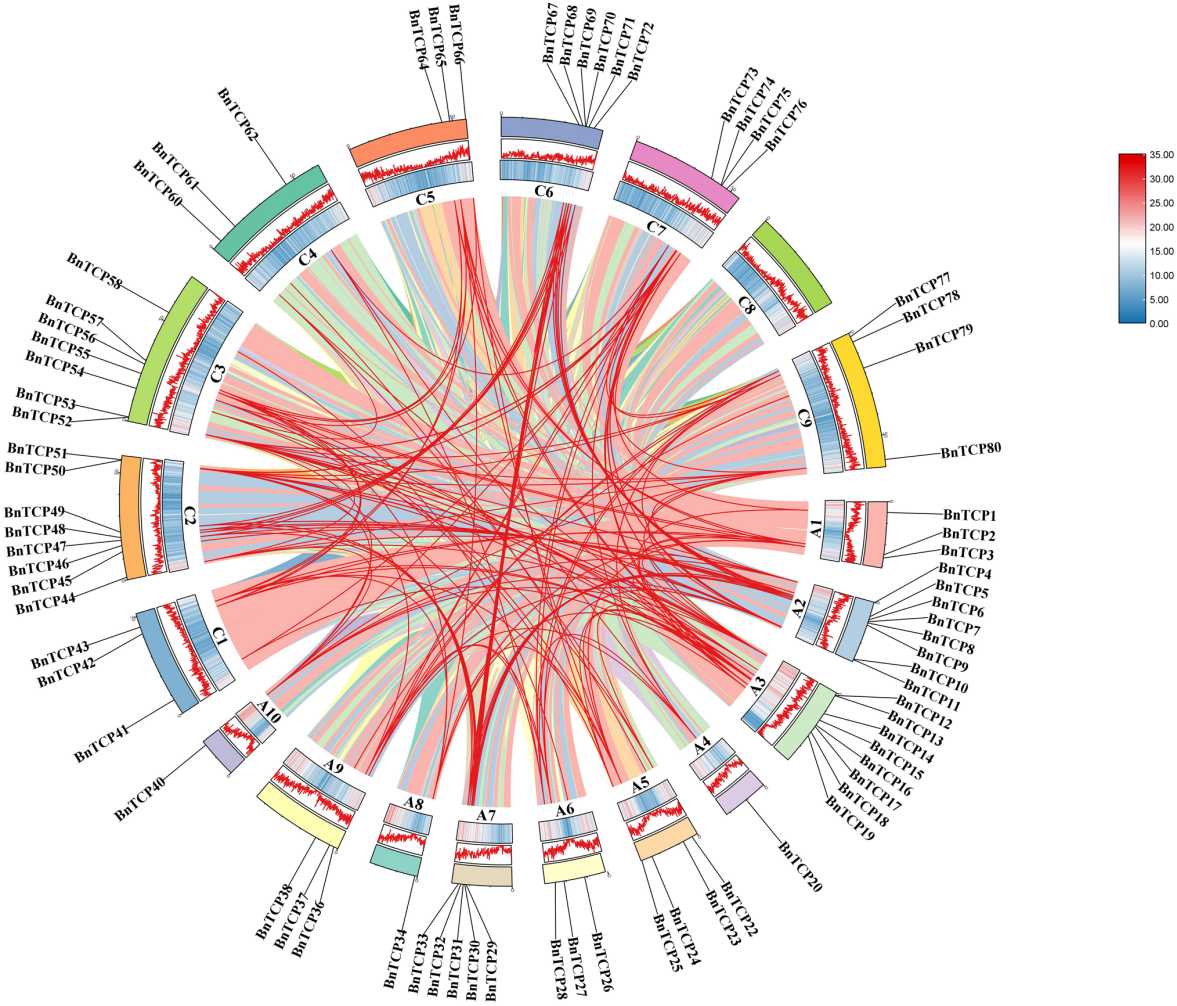
Schematic representation of the chromosomal distribution and interchromosomal relationships of *B. napus TCP* genes. Colored lines indicate all synteny blocks in the *B. napus* genome, and the red lines indicate duplicated *TCP* gene pairs. The chromosome number is indicated at the bottom of each chromosome

Polyploidization, interspecies hybridization, or whole-genome duplication (WGD) is an important evolutionary phenomenon that has occurred multiple times in all angiosperms over millions of years (Das et al. 2020; Fan et al. 2024). In addition, small-scale events such as tandem or fragment duplication and inversion also enable plants to reshape their genomes. We did not observe tandem duplication events in the *TCP* gene family in rapeseed (Fig. 3). However, a large number of fragment duplication genes were identified, indicating that fragment duplication may have contributed to the expansion of the *TCP* gene family and revealing a significant occurrence of *BnTCP* genes. A total of 159 pairs of segmental duplications, representing duplications of large chromosomal regions, were detected (Fig. 4, Table S5). Chromosome A3 had the highest *BnTCP* membership (*n* = 35), while chromosome A4 had the lowest (*n* = 5). Among the identified *BnTCP* genes, 47 segmental duplications were detected in subfamily CIN, 27 segmental duplications were detected in family CYC/TB1, and 85 segmental duplications were detected in family PCF. A total of 85 pairs (~ 53.45%) of repetitive events involving 37 genes (~ 46.25%) belong to the subfamily PCF. A total of 85 pairs of repetitive events involving 37 genes belong to the subfamily PCF. In addition, seven genes (*BnTCP10*, *BnTCP13*, *BnTCP40*, *BnTCP50*, *BnTCP80*, *BnTCP53*, and *BnTCP74*), all of which belong to the subfamily PCF, exhibited eight or nine homologous expansion events. This, to some extent, confirms why PCFs constitute the largest subfamily of the *TCP* family in rapeseed. These gene replication events contributed to the expansion of numerous *BnTCP* members, potentially playing a pivotal role in evolutionary processes and the acquisition of new functions related to environmental adaptation within the *TCP* gene family in *B. napus*.

### Synteny analysis of *BnTCP* genes

**Fig. 5:**
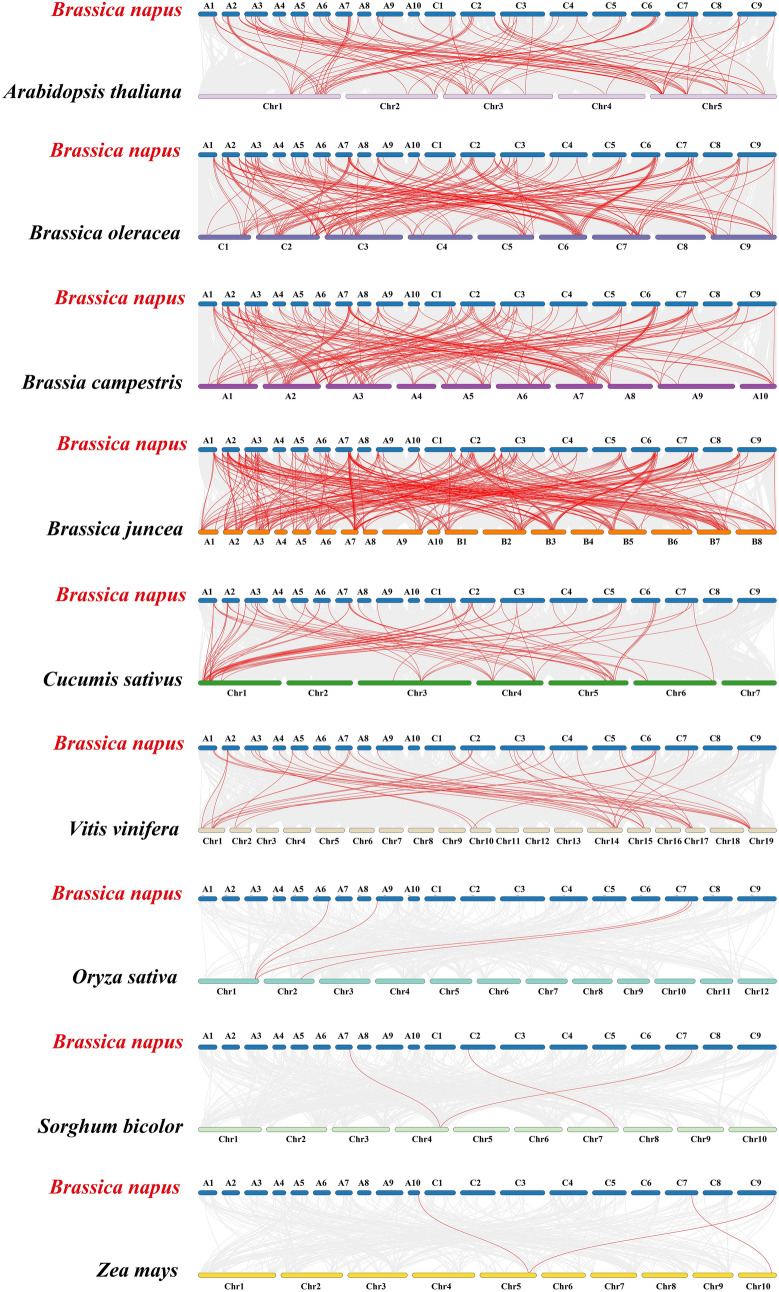
Synteny analyses of the *TCP* genes between *B. napus* and nine representative plant species (*S. bicolor*, *O. sativa*, *Z. mays*, *A. thaliana*, *C. sativus*, *V. vinifera*, *B. rapa*, *B. oleracea*, and *B. juncea*). Gray lines on the background indicate the collinear blocks in *B. napus* and other plant genomes; red lines highlight the syntenic *B. napus TCP* gene pairs

To clarify the evolutionary connections among TCP proteins across diverse plant species, synteny maps were generated utilizing homologous genes from *B. napus* and nine other representative plant species. The selected species included three monocotyledonous plants (*Sorghum bicolor*, *Oryza sativa*, *Zea mays*) and six dicotyledonous plants (*A. thaliana*, *Cucumis sativus*, *Vitis vinifera*, *B. rapa*, *B. oleracea*, and *B. juncea*) (Fig. 5, Table S6). The 69 BnTCP proteins exhibited homologous relationships with proteins in *A. thaliana* (68 genes), *O. sativa* (4), *Z. mays* (3), *S. bicolor* (3), *V. vinifera* (36), *C. sativus* (42), *B. rapa* (68), *B. oleracea* (67), and *B. juncea* (69), as summarized in Table S6. The numbers of collinear gene pairs between *B. napus* and other representative species (*O. sativa*, *Z. mays*, *S. bicolor*, *A. thaliana*, *V. vinifera*, *C. sativus*, *B. rapa*, *B. oleracea*, and *B. juncea*) were 97, 4, 3, 43, 187, 191, 299, and 73, respectively. Among the monocotyledonous plants (*S. bicolor*, *O. sativa*, and *Z. mays*), only one homologous gene, *BnTCP74*, was identified, suggesting the preservation of these homologous genes over long-term evolution, possibly predating the differentiation of ancestral plants.

Furthermore, a substantial number of collinear gene pairs, consisting of 68 BnTCP proteins, were identified between *B. napus* and dicotyledonous plants. In the six dicotyledonous plants, each exhibited at least one pair of homologous genes. Notably, five proteins (BnTCP20, BnTCP24, BnTCP34, BnTCP36, and BnTCP40) contained four pairs of homologous members, indicating stronger amplification in dicotyledonous plants. In cruciferous plants, ten protein members (BnTCP14, BnTCP20, BnTCP23, BnTCP24, BnTCP26, BnTCP34, BnTCP36, BnTCP37, BnTCP38, and BnTCP40) exhibited three homologous pairs, suggesting stronger amplification in cruciferous plants. To discern the targeted or balanced selection among the 80 BnTCP proteins, Tajima-D neutrality testing was conducted, and the results are presented in Table S7. The calculated D value of 8.95 signifies a significant deviation from 0, suggesting that the *BnTCP* gene family likely plays a role in the evolution of neutral selection.

### Evolutionary analysis of the *BnTCP* and *TCP* genes of several different species

**Fig. 6:**
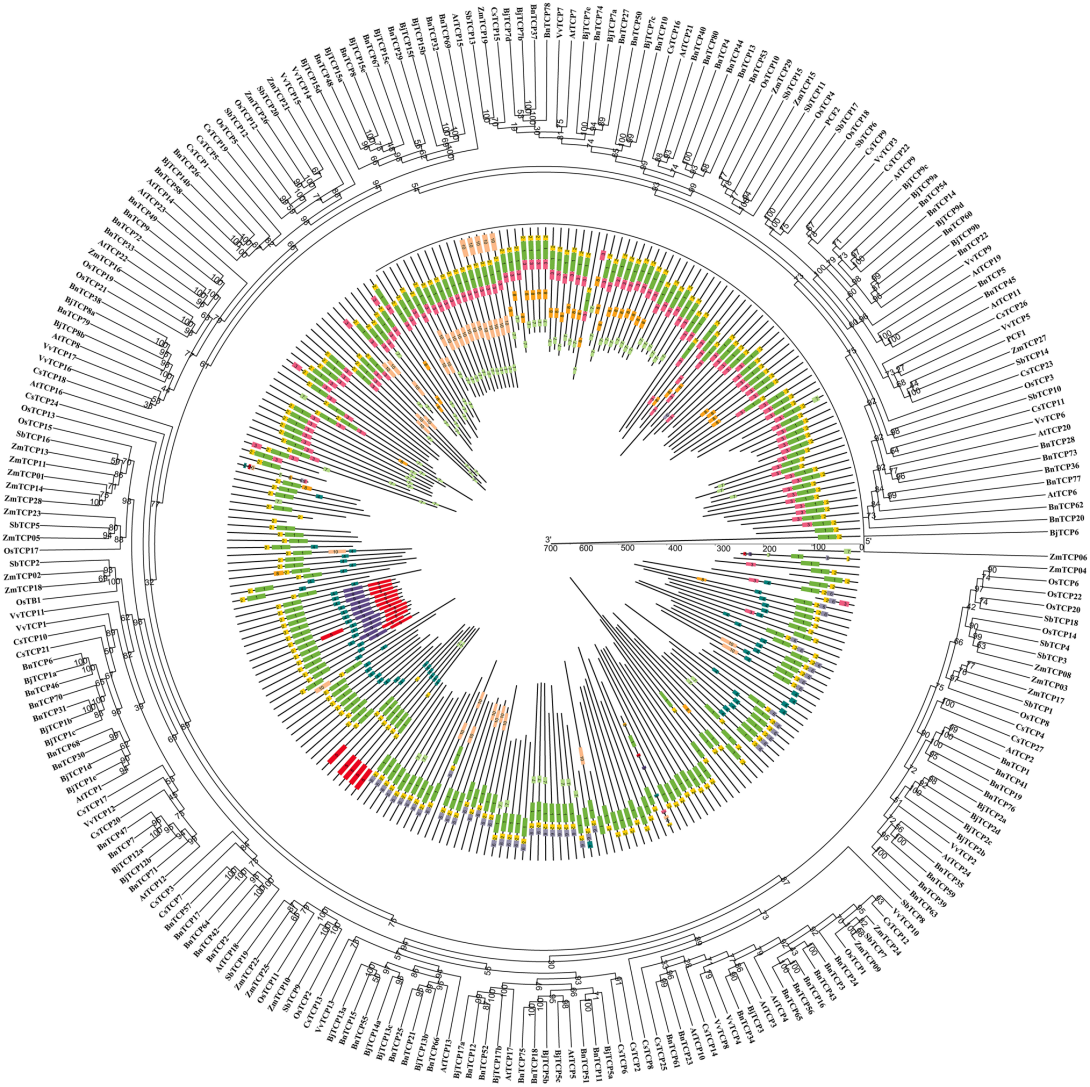
Phylogenetic relationship and motif composition of the TCP proteins from *B. napus* with nine representative plant species (*S. bicolor*, *O. sativa*, *Z. mays*, *A. thaliana*, *C. sativus*, *V. vinifera*, *B. rapa*, *B. oleracea*, and *B. juncea*). Outer panel: an unrooted phylogenetic tree constructed using Geneious R11 with the neighbor-joining method. Inner panel: distribution of conserved motifs in TCP proteins. The differently colored boxes represent different motifs and their positions in each TCP protein sequence

To examine the genetic relationships of TCP proteins between *B. napus* and nine representative plants (*O. sativa*, *Z. mays*, *S. bicolor*, *A. thaliana*, *V. vinifera*, *C. sativus*, *B. rapa*, *B. oleracea*, and *B. juncea*), we constructed an unrooted neighbor-joining tree. Using the MEME online service software, we identified 10 conserved motifs in the sequences of 266 BnTCP proteins from these plants (Fig. 6, Table S3). The detailed motifs are outlined in Tables S2 and S3. BnTCP proteins tend to cluster with TCP members of dicotyledonous plants, particularly in *A. thaliana*, *B. rapa*, *B. oleracea*, and *B. juncea*. All remaining BnTCP proteins were characterized by motif 1. In general, the TCP proteins of dicotyledonous plants and *B. napus* on the same topological branch exhibit similar motif arrangements. Within these plants, specific TCP protein subfamilies frequently share similar motifs, suggesting their evolutionary relationships. Notably, motifs 1, 3, and 9 collectively form a conserved structure, predominantly aggregating within the CYC/TB1 subfamily, while motifs 1, 2, and 3 tend to cluster within the PCF subfamily. In summary, the coherence of class organization was robustly supported through the assessment of conserved motif composition, gene structures, and phylogenetic interactions. These analyses suggest that TCP proteins exhibit remarkably preserved amino acid residues and that members within a class may play analogous functional roles. This finding underscores the intricate molecular characteristics and potential functional similarities among these same subfamilies.

### Expression patterns of *BnTCP* in several plant organs

**Fig. 7:**
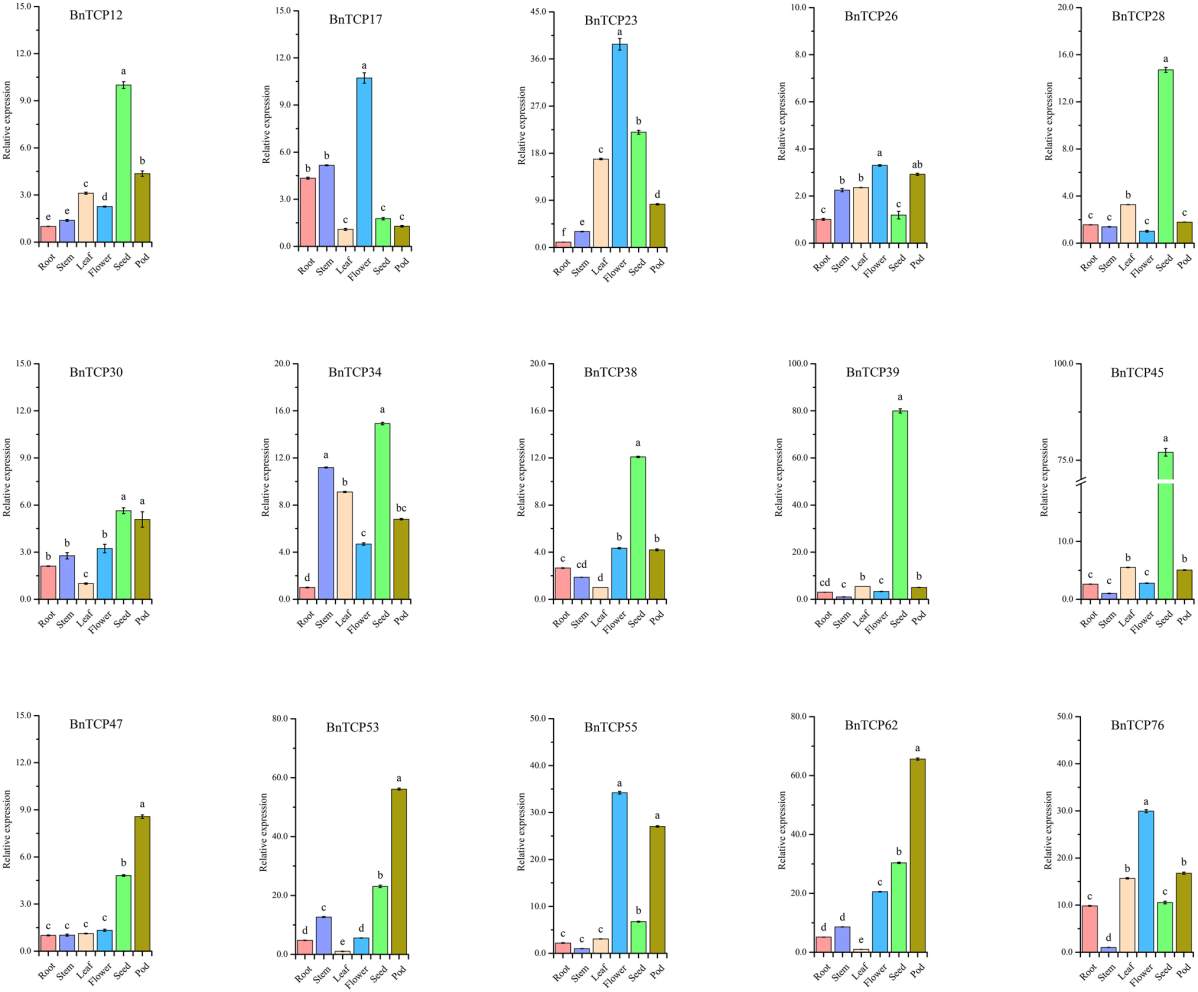
Expression patterns of 15 *B. napus TCP* genes in the roots, stems, leaves, flowers, seeds, and peels were examined via qRT-PCR. Error bars are obtained from three measurements. The SE is selected as the value of the bar. Lowercase letters above the bars indicate significant differences (α = 0.05, LSD) among the treatments. The same below

To elucidate the physiological roles of the 80 *TCP* genes in *B. napus*, we employed quantitative real-time PCR (qRT‒PCR) to assess the expression profiles of 15 selected *BnTCP* genes (Fig. 7). Specifically, members from distinct subfamilies were chosen based on marked variations in amino acid sequences and phylogenetic relationships. We quantified the transcript accumulation of these *BnTCP* genes across six organs—leaf, stem, root, flower, seed, and peel—as illustrated in Fig. 7. Numerous *BnTCP* genes exhibited tissue-specific expression patterns in *B. napus*. Notably, five genes (*BnTCP17*, *BnTCP23*, *BnTCP26*, *BnTCP55*, and *BnTCP76*) exhibited peak expression in flowers. Conversely, seven genes (*BnTCP12*, *BnTCP28*, *BnTCP30*, *BnTCP34*, *BnTCP38*, *BnTCP39*, *BnTCP45*) were predominantly expressed in the seeds, and six genes (*BnTCP26*, *BnTCP30*, *BnTCP47*, *BnTCP53*, *BnTCP55*, *BnTCP62*) exhibited maximal expression in the peels. The expression of one gene, *BnTCP34*, was highest in the stems. The expression pattern of the *BnTCP* gene is coordinated in several plant organs, indicating that their effects may be synergistic (Fig. S1). The *TCP* gene showed a significant positive correlation. For example, four genes (*BnTCP30*, *BnTCP47*, *BnTCP53*, and *BnTCP62*) were significantly positively correlated. *BnTCP12*, *BnTCP28*, *BnTCP38*, *BnTCP39*, and *BnTCP45* also exhibited significant positive correlations. However, there was a significant negative correlation between many genes related to the *BnTCP* genes (*BnTCP26* and *BnTCP28/BnTCP39/BnTCP45*).

**Fig. 8:**
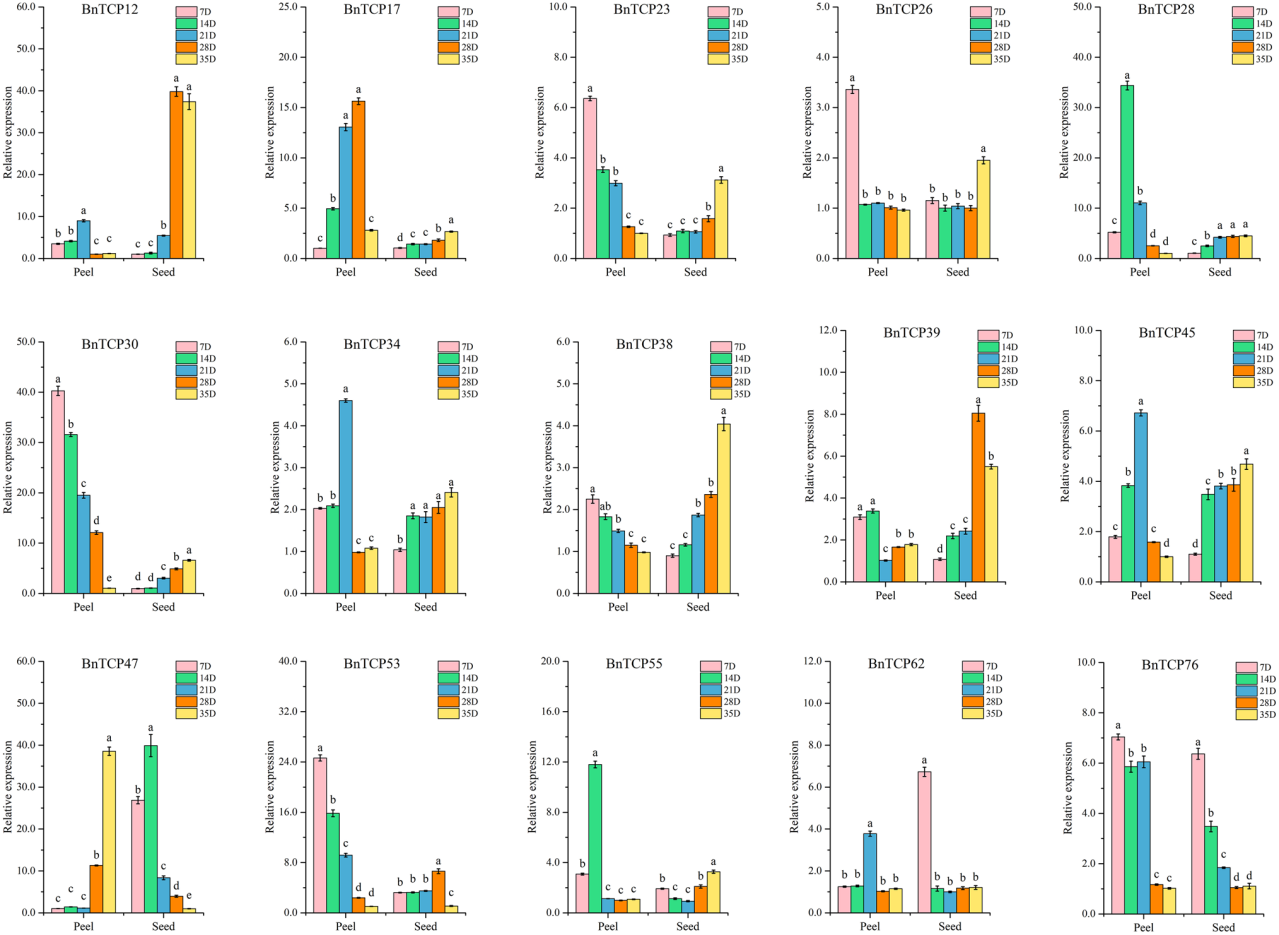
Expression patterns of 15 *B. napus TCP* genes were examined during different fruit development stages: 7 DPA (early filling stage), 14 DPA (mid filling stage), 21 DPA (early ripening stage), 28 DPA (mid ripening stage), and 35 DPA (full ripening stage)

Certain *BnTCP* genes may play pivotal roles in modulating seed development in *B. napus*, consequently influencing its nutritional profile and developmental pace. To identify potential regulatory genes associated with *B. napus* development, we examined the expression patterns of 15 *BnTCP* genes in seeds and peels (Fig. 8). Notably, the expression levels of most *BnTCP* genes in fruits and peels varied across the five developmental stages. We discerned three distinct expression patterns across different developmental phases, characterized by genes whose expression either increased, decreased, or initially increased before decreasing. Specifically, in peels, seven genes (*BnTCP23*, *BnTCP26*, *BnTCP30*, *BnTCP38*, *BnTCP39*, *BnTCP53*, and *BnTCP76*) exhibited peak expression at 7 DPA, while five genes (*BnTCP12*, *BnTCP17*, *BnTCP34*, *BnTCP45*, and *BnTCP62*) reached their peak expression levels at 21 DPA. In the seeds, the expression profiles of 10 genes, namely, *BnTCP12*, *BnTCP17*, *BnTCP23, BnTCP26*, *BnTCP28*, *BnTCP30*, *BnTCP34*, *BnTCP38*, *BnTCP45*, and *BnTCP55*, peaked at 35 DPA. Conversely, five genes, specifically *BnTCP12*, *BnTCP28*, *BnTCP34*, *BnTCP39*, and *BnTCP53*, exhibited their highest expression levels at 28 DPA. This temporal variation in gene expression suggests dynamic regulatory mechanisms during seed development in *B. napus*. During fruit development, some gene modules exhibit synergistic/antagonistic expression (Fig. S2). For instance, *BnTCP12*, *BnTCP34*, *BnTCP45*, and *BnTCP62* exhibited significantly positively correlated expression patterns. However, *BnTCP23*, *BnTCP30*, *BnTCP38*, *BnTCP53*, and *BnTCP76* formed significantly positively correlated expression modules but were negatively correlated with the expression pattern of *BnTCP47*.

To investigate potential alterations in the transcription patterns of *BnTCP* members in *B. napus* under various abiotic stress conditions, the plants were subjected to four distinct stressors: polyethylene glycol (PEG) at 10%, sodium chloride (NaCl) at 5%, heat stress at 40 °C, and cold stress at 4 °C. Using quantitative real-time polymerase chain reaction (qRT‒PCR), the expression profiles of 15 *BnTCP* genes were evaluated across roots, leaves, and stems in response to various abiotic stresses (Fig. S3). The findings indicate that numerous *BnTCP* members exhibit significant upregulation or inhibition in the face of distinct stressors. This sensitivity of most *TCP* genes to environmental changes leads to pronounced differences in their expression levels under short-term stress, suggesting their potential involvement in stress regulatory mechanisms. For instance, under NaCl stress, the expression of *BnTCP26*, *BnTCP30*, and *BnTCP38* significantly increased across roots, stems, and leaves within 1 h. Notably, the expression patterns of *BnTCP* genes evolve over time in different organs, contingent upon the specific stress treatment. Specifically, under heat stress, *BnTCP17*, *BnTCP39*, *BnTCP55*, and *BnTCP76* in stems were initially upregulated and subsequently downregulated. Conversely, *BnTCP12* exhibited a gradual and significant downregulation in stems but a concurrent upregulation in leaves under PEG stress. Intriguingly, several *BnTCP* genes display opposing expression patterns under distinct abiotic stresses. For instance, *BnTCP12* was upregulated in response to cold stress but downregulated under PEG stress. Moreover, a subset of *BnTCP* genes demonstrated analogous expression patterns across various stress treatments. Notably, *BnTCP30* and *BnTCP47* exhibited increasing expression in roots under diverse stress conditions. These observations underscore the intricate regulatory network involving *BnTCP* genes in response to different abiotic stresses, shedding light on their potential roles in stress adaptation mechanisms in *B. napus*. Furthermore, a positively correlated response pattern was observed for a total of ten *BnTCP* genes (Fig. S4). This positive correlation in the expression profiles of these genes adds an intriguing layer to our understanding of their functional relationships or coordinated regulation in the studied context. The synchronized behavior of these specific *BnTCP* genes hints at a potential shared regulatory network or common signaling pathways influencing their expression. This finding contributes valuable insights into the intricate molecular dynamics governing the coordination of these genes and underscores their potential significance in the biological processes under investigation.

The *TCP* family in *B. napus* is hypothesized to play a role in both abiotic stress responses and hormone signaling. Consequently, we investigated the expression patterns of these *TCP* family members under various hormone treatments (Figs. S5 and S6). Four pivotal hormones—gibberellin, auxin, jasmonic acid, and abscisic acid—were chosen for analysis. The resulting expression data were utilized to construct a correlation network, as illustrated in Fig. S6. Overall, some genes exhibited synergistic expression patterns. For instance, *BnTCP28* and *BnTCP76* were coexpressed under gibberellin induction, while *BnTCP26* and *BnTCP53* were coexpressed under jasmonic acid induction. Conversely, certain genes displayed contradictory expression patterns. Notably, the expression pattern of *BnTCP12* exhibited a significant positive correlation with that of *BnTCP55* and *BnTCP62* but a negative correlation with that of *BnTCP28* and *BnTCP45* following short-term treatment with abscisic acid. This intricate interplay underscores the functional complexity within different subfamily members, likely tied to the diversity of promoter elements and amino acid structures. Nevertheless, further empirical evidence is required to validate these hypotheses.

## Discussion

### *BnTCP* gene structure and evolutionary analysis

An exhaustive analysis of the *TCP* gene family in rapeseed has been conducted, yielding a total of 80 *BnTCP* genes. These proteins display considerable structural diversity, highlighting the complex and nuanced nature of the *TCP* family. (Huang et al. 2022). The length of TCP proteins ranges from 201 to 538 amino acids, manifesting substantial variation in the length and sequence composition of *TCP* genes (Cubas et al. 1999a, b; Huang et al. 2022). The ratio of *BnTCP* genes to the total number of genes in the *B. napus* genome was approximately 0.06%, a value lower than that observed in *Arabidopsis* (0.26%) (Yao et al. 2007), *N. tabacum* (0.06%) (Chen et al. 2016), *B. juncea* (0.15%) (He et al. 2020), and *Z. mays* (0.07%) (Chai et al. 2017) but higher than that in *S. bicolor* (0.05%) (Zheng et al. 2019) and *B. oleracea* (0.05%) (Liu et al. 2018a, b). Phylogenetic analysis categorized the TCP proteins of rapeseed into 3 subfamilies: CIN, PCF, and CYC/TB1 (Fig. 1). The presence of at least one BnTCP protein corresponding to each *Arabidopsis TCP* subfamily suggests the long-standing conservation of these subfamilies across extensive evolutionary timescales, implying that they may play essential and conserved roles in fundamental biological processes (Chai et al. 2017; Efroni et al. 2008). This observation underscores the stability and importance of *TCP* subfamilies in the evolutionary landscape, as manifested by their presence across both *B. napus* and *Arabidopsis*. The retention of these proteins in different subfamilies suggests a degree of functional indispensability that has persisted over evolutionary timescales. Consequently, the unique physiological roles of these BnTCP proteins warrant further experimental investigation. Within these subfamilies, the PCF subfamily exhibited the greatest number of members (38, ~ 47.5%), while the CYC/TB1 subfamily had the fewest members (14, ~ 17.5%). Similar to *the TCP* subfamilies in *Arabidopsis* (Yao et al. 2007), rice (Yao et al. 2007; Danisman et al. 2016), buckwheat (Yang et al. 2022), and sorghum (Zheng et al. 2019), these distinct subfamilies may exhibit diverse capacities for differentiation over the course of long-term evolution. The evolutionary divergence among these genes is likely instrumental in their performing a range of functions across various species. Nonetheless, there is a paucity of research investigating the potential interplay between environmental adaptation and the differentiation within *TCP* subfamilies.

The percentage of *BnTCP* genes lacking introns (35, ~ 43.8%) was lower than that observed in rice (55%) (Danisman et al. 2016), Tartary buckwheat (41, ~ 87%) (Yang et al. 2022), and sorghum (66.67%) (Zheng et al. 2019). Similar occurrences of genes devoid of introns are noted in other extensive gene families, exemplified by the F-box transcription factor gene family (Jain et al. 2007) and DEAD-box RNA helicase (Aubourg et al. 1999). In addition to replication events, the relationships and numbers of exons and introns contribute to elucidating the evolutionary narrative of gene families (Xu et al. 2012; Raza et al. 2021; Su et al. 2021). The analysis of the *BnTCP* gene structures revealed a consistent exon/intron distribution in terms of both exon and intron numbers within the *TCP* gene family. Moreover, members of the same *BnTCP* subfamily in grous exhibit analogous exon/intron structures. The results compellingly indicate that these *TCP* members likely assume comparable roles in the response to a spectrum of abiotic stresses. It is noteworthy that analogous patterns have been documented in prior research on *TCP* gene families, where specific intron/exon configurations and motif arrangements were identified across different gene types (Lin et al. 2016; Yin et al. 2018). *TCP* genes potentially trace their origins to prokaryotic genes via horizontal gene transfer, resulting in repeated events during evolutionary processes. Consequently, a significant proportion of *TCP* gene family members exist as genes lacking introns (Liu et al. 2022). In an evolutionary context, the presence of introns within genes can augment gene length and the frequency of intergene recombination, thereby positively influencing evolution (Shabalina et al. 2010). However, intron-free genes do not remain secluded during transcription and translation processes; they sustain continuous protein encoding and display a propensity for swift responses to environmental changes (Sang et al. 2016; Jain et al. 2008).

Fragment replication events play pivotal roles in the expansion of the *TCP* gene family in rapeseed. We identified 159 pairs of fragment duplications within the *BnTCP* gene family, encompassing 75 members (~ 93.8%). The majority of these events originated from the PCF subfamily, suggesting a heightened preference for replication events among certain *TCP* subfamily members. Notably, these duplicated genes do not exhibit significant structural disparities postreplication events (Ma et al. 2014). Tandem repeat events were not detected within the *BnTCP* gene family, emphasizing the contribution of fragment repetition events to the expansion of *TCP* members in rapeseed. This pattern aligns with observations in Tartary buckwheat (Yang et al. 2022) and sorghum (Zheng et al. 2019), suggesting a broader prevalence of the PCF subfamily in plant evolution, extending beyond monocotyledonous plants. Gene duplication events within *TCP* genes have also been identified in various plant species (İlhan et al. 2018; Mondragón-Palomino et al. 2011; Wang et al. 2022; Zhao et al. 2021). A comparative analysis within the same group demonstrated that the architecture of *BnTCP* genes is characterized by a conserved exon/intron arrangement, both in terms of exon and intron counts. In addition, genes within the same *BnTCP* subgroup displayed analogous motif compositions. These data imply that the *TCP* members in question likely perform similar functions in the response to a variety of abiotic stressors. The uniformity observed in exon/intron distribution and motif arrangements is reminiscent of previous studies on *TCP* gene families, where distinct groups have been found to exhibit parallel patterns of intron/exon arrangement and motif composition (İlhan et al. 2018; Mondragón-Palomino et al. 2011; Wang et al. 2022; Zhao et al. 2021).

### Expression patterns and functional prediction of* BnTCP*

*BnTCP26* exhibited the highest expression in flowers, aligning with the expression pattern of its homologous gene *AT3G47620* (Han et al. 2019). *AT3G4762*0 is suggested to play a crucial role in inflorescence development and seed germination in *Arabidopsis*. The transcription levels of both *BnTCP45* and its homologous gene *AT5G51910* were notably elevated in seeds, with *AT5G51910 being* implicated in the developmental transition from the vegetative to embryonic stage (Zhang et al. 2019). As a member of the CIN subfamily, *BnTCP12* displayed heightened expression in plant seeds, indicating its significant role in the growth and development of rapeseed. Conversely, *BnTCP34*, another member of the CIN subfamily, exhibited a greater expression level in stems. Within the CIN subfamily, *BnTCP55* displayed elevated expression levels in both flowers and peels, suggesting distinct expression patterns among genes within the same subfamily. *BnTCP17* of the CYC/TB1 subfamily demonstrated high expression in flowers, underscoring its potential importance, although further specific functions necessitate in-depth experimental analysis. Among the other CYC/TB1 subclasses, *LjCYC1* and *LjCYC3* in *Lotus japonicus* and *AmCYC* in *Antirrhinum majus* have significant effects on flower development (Luo et al. 1996; Wang et al. 2010). In addition, *AtTCP1* and *GhCYC2* from *Gerbera jamesonii* control the growth of symmetrical petals in flowers (Koyama et al. 2010a, b; Juntheikki-Palovaara et al. 2014). *ZmTB1* in maize, *AtBRC1* and *AtBRC2* in *A. thaliana* and *OsTB1* in rice regulate branches by negatively regulating axillary bud growth (Aguilar-Martínez et al. 2007; Takeda et al. 2003). In addition, *TCPs* regulate the biological clock and plant morphogenesis. *AtTCP2*, *AtTCP*3, *AtTCP11* and *AtTCP15* combine with the TGGGC (C/T) element and interact with various components of the core circadian rhythm, thus adjusting the circadian clock in *Arabidopsis* (Wu et al. 2016). Futhermore, *BnTCP30* and *BnTCP47*, which are also members of the CYC/TB1 subfamily, exhibited increased expression in seeds and peels. This indicates that the physiological functions of members of the subfamily CYC/TB1 may be complex. The expression of PCF subfamily genes was detected in both the seeds and peels, suggesting that these genes have a significant impact on these plant parts. For instance, *BnTCP26* exhibited the highest expression levels in both seeds and peels, and three genes (*BnTCP28*, *BnTCP38*, and *BnTCP45*) displayed heightened expression in seeds. In addition, we observed that the expression level of *BnTCP76* was greatest in both the seeds and peels at 7 DPA but decreased over time. Conversely, the expression levels of *BnTCP23*, *BnTCP30*, and *BnTCP38* in the peel decreased continuously with time, while their expression levels in the seeds increased progressively. These findings provide insights into the dynamic expression patterns of *BnTCP* genes in different plant tissues over time. As an important oil plant, rapeseed may regulate its adaptation to the environment through complex endogenous networks and transcription signals, and similar conclusions have been drawn for *Arabidopsis*, grape and sorghum. Previous studies have shown that members of the same subfamily with the same motif may have similar physiological functions. Therefore, we can further speculate on the function of the *BnTCP* genes, but further experiments are needed for verification.

As an important oil plant, rape may regulate its adaptation to the environment through complex endogenous networks and transcription signals, and similar conclusions have been drawn for *Arabidopsis*, grape and sorghum. Previous studies have consistently demonstrated that members of the same subfamily within the *TCP* gene family, which share conserved motifs, are likely to possess analogous physiological functions. Consequently, it is plausible to hypothesize the functional roles of *BnTCP* genes based on these patterns. However, to substantiate these hypotheses, additional experimental evidence is imperative. The upregulation of *BnTCP30* expression in rapeseed roots under cold stress potentially enhances the environmental adaptability of rapeseed plants. The expression of *BnTCP12*, a member of the CIN subfamily, significantly increases in the roots, stems, and leaves of plants subjected to stressors, such as NaCl, heat, and cold. *AT5G08070* plays a pivotal role in promoting thermal morphology, yet further validation through subsequent experiments is warranted (Danisman et al. 2013). Treatment with four hormones (ABA, IAA, GA, and MeJA) increased the expression of numerous genes, consistent with recent findings. For instance, in cotton, 41 *GhTCP* genes exhibit substantial responses to heat, salinity, and drought stress (Yin et al. 2018). In cucumber, select *TCP* genes are upregulated under photoperiod and temperature stress (Wen et al. 2020). Similarly, some genes are induced upon exposure to plant hormones, such as ethylene and gibberellin. In *Phyllostachys pubescens*, certain *TCP* genes are significantly upregulated in response to ABA, SA, and other hormones (Liu et al. 2018a, b). Overexpression of the maize *ZmTCP42* gene in *Arabidopsis* leads to enhanced sensitivity of seed germination to ABA and improved drought stress tolerance (Ding et al. 2019). In a separate study, overexpression of the *MeTCP4* gene was shown to regulate cold tolerance by mediating ROS production and elimination in transgenic *Arabidopsis*. It also elevates the expression levels of stress- and ROS scavenging-related genes under cold stress in *Arabidopsis* (Cheng et al. 2019). Overexpressing the *PeTCP10* gene enhances salt and ABA tolerance during the vegetative growth period of transgenic *A. thaliana* (Xu et al. 2021). These findings collectively emphasize the pivotal role of *TCP* genes in facilitating hormone signaling pathways and augmenting abiotic stress resilience across a broad spectrum of plant species.

## Conclusion

This study offers a thorough genomic analysis of the *BnTCP* gene family in *Brassica napus* (rapeseed). The family comprises 80 members, which have been systematically categorized into three subfamilies. We have undertaken an extensive examination of their gene structure, evolutionary relationships, conserved motifs, gene duplication events, and expression profiles. Significantly, our investigation revealed a positive correlation between fragment repetition and the amplification of the *BnTCP* family, highlighting the potential significance of fragment repetition in the evolutionary expansion of *TCP* genes in rapeseed. Moreover, we conducted a detailed examination of the expression dynamics of *BnTCP* genes across various tissues, time periods, and responses to diverse stresses and hormonal influences. Specific genes, such as *BnTCP12*, *BnTCP34*, and *BnTCP76*, were identified for their pronounced relevance to tissue development and responses to environmental stressors. These findings provide valuable insights that contribute to a more nuanced understanding of the functional roles played by the *TCP* gene family in rapeseed.

## Supplementary Information

Below is the link to the electronic supplementary material.

Supplementary file1 (JPG 19944 KB)

Supplementary file2 (JPG 2099 KB)

Supplementary file3 (JPG 20391 KB)

Supplementary file4 (JPG 2095 KB)

Supplementary file5 (XLSX 10 KB)

Supplementary file6 (XLS 157 KB)

Supplementary file7 (XLS 23 KB)

Supplementary file8 (XLSX 1086 KB)

Supplementary file9 (XLSX 17 KB)

Supplementary file10 (XLS 179 KB)

Supplementary file11 (XLSX 10 KB)

Supplementary file12 (JPG 2060 KB)

Supplementary file13 (JPG 2112 KB)

## Data Availability

The entire *B. napus* genome sequence information was obtained from the National Center for Biotechnology Information (NCBI) GenBank website (accession number GCF_020379485.1). The rapeseed materials (Tianfuyouchun Bn2029) used in the experiment were supplied by Researcher Pu Xiaobin, Crop Research Institute, Sichuan Academy of Agricultural Sciences. The data sets supporting the conclusions of this study are included in the article and its additional files.
