# Supplementary figures and images for: Genome-wide identification and expression analysis of the TCP transcription factor family and its response to abiotic stress in rapeseed (Brassica napus L.)
Source: 3 Biotech. 2025 Apr 7;15(5):119. doi: 10.1007/s13205-025-04273-x (PMC11977093; doi:10.1007/s13205-025-04273-x)

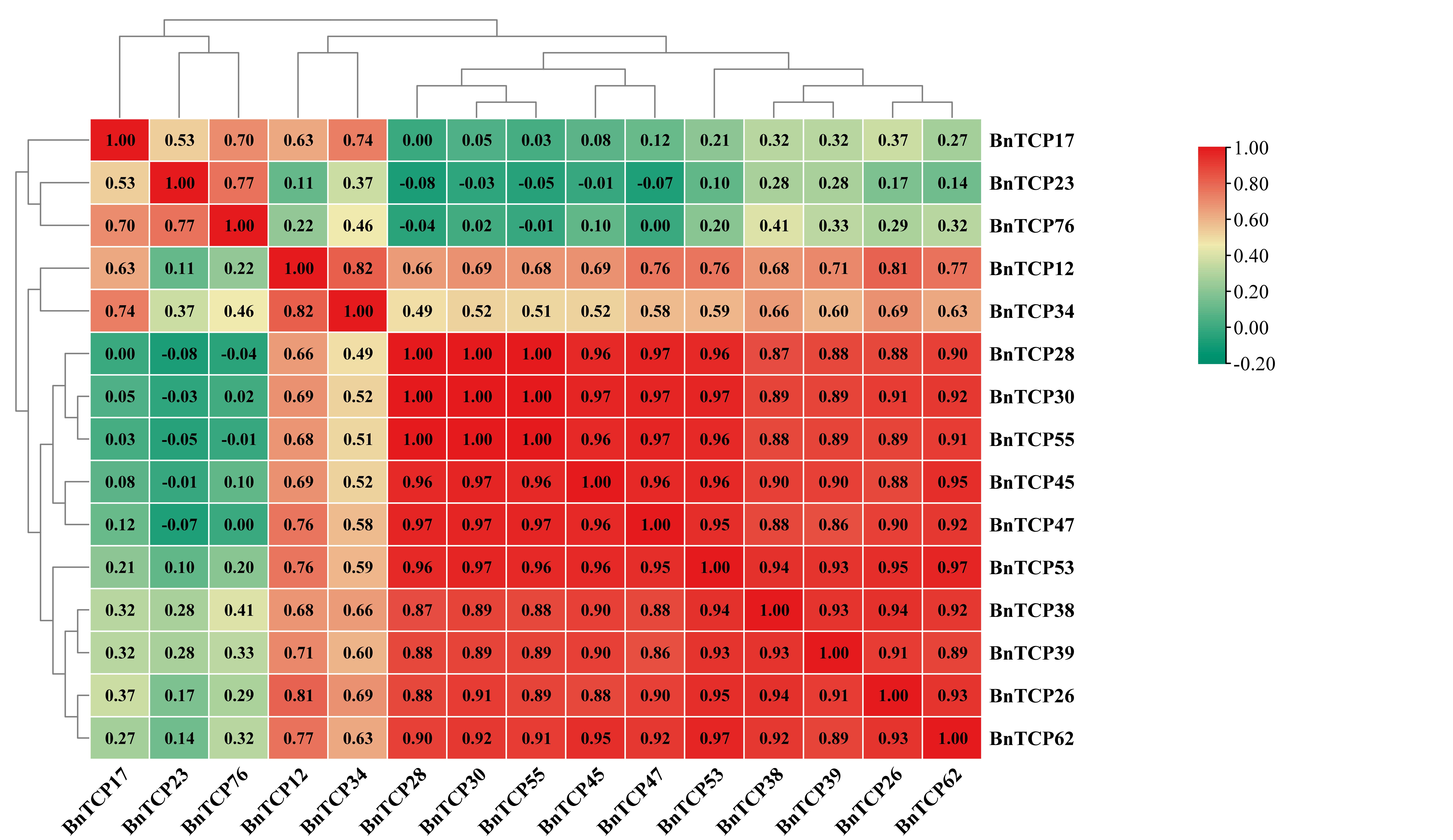

Supplement: Supplementary file 2 — Supplementary file2 (JPG 2099 KB) [file 13205_2025_4273_MOESM2_ESM.jpg]

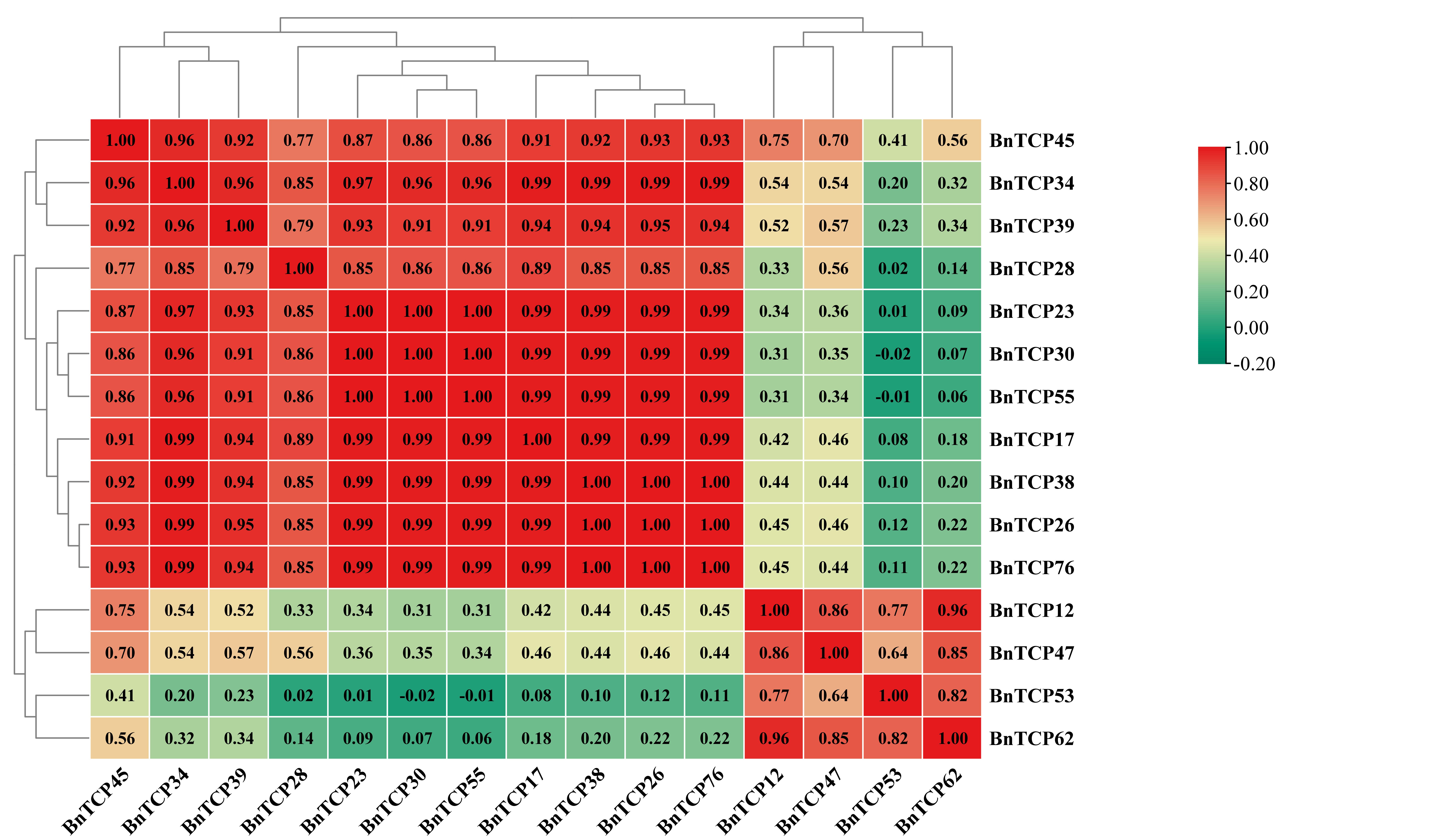

Supplement: Supplementary file 4 — Supplementary file4 (JPG 2095 KB) [file 13205_2025_4273_MOESM4_ESM.jpg]

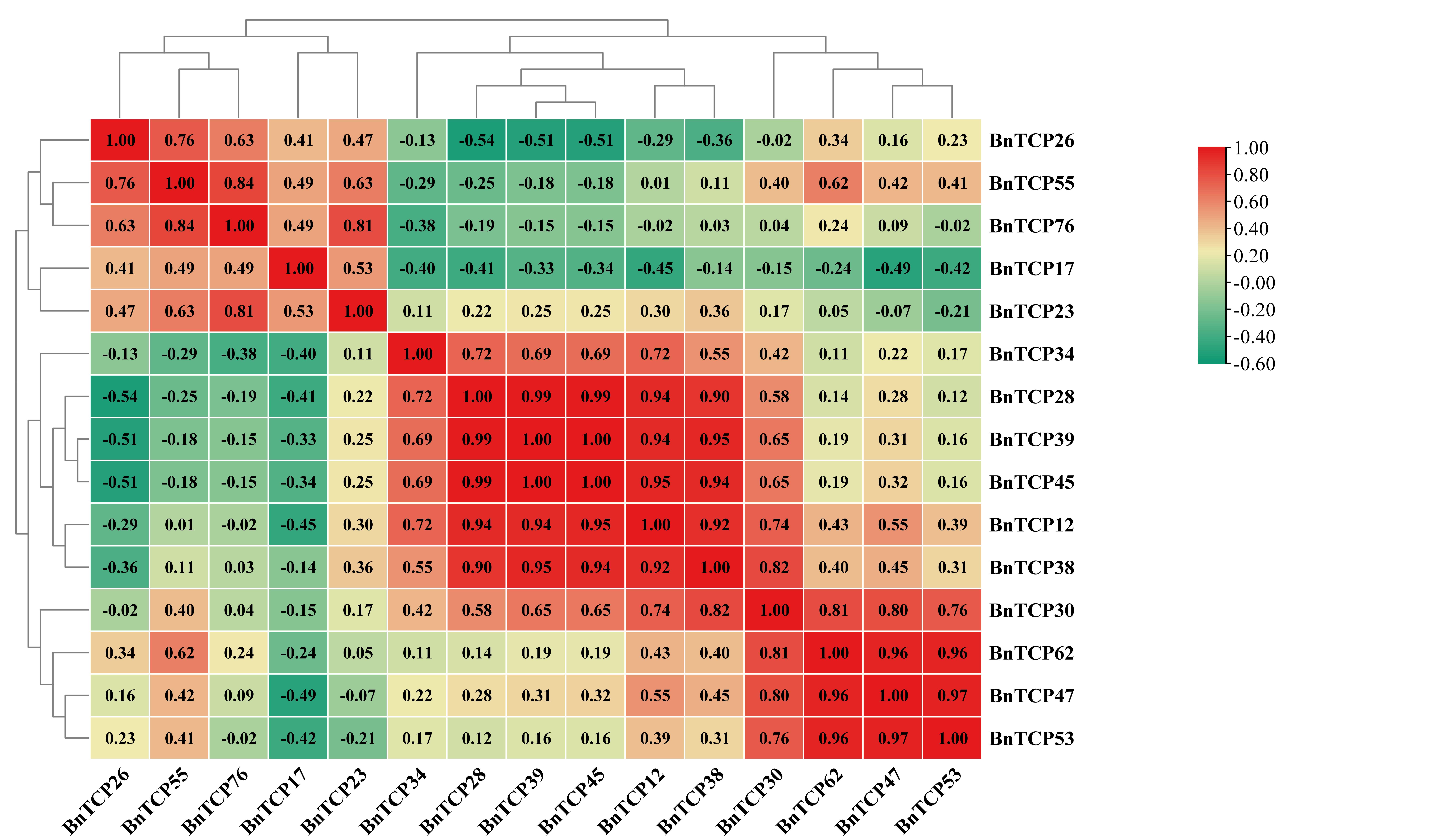

Supplement: Supplementary file 12 — Supplementary file12 (JPG 2060 KB) [file 13205_2025_4273_MOESM12_ESM.jpg]

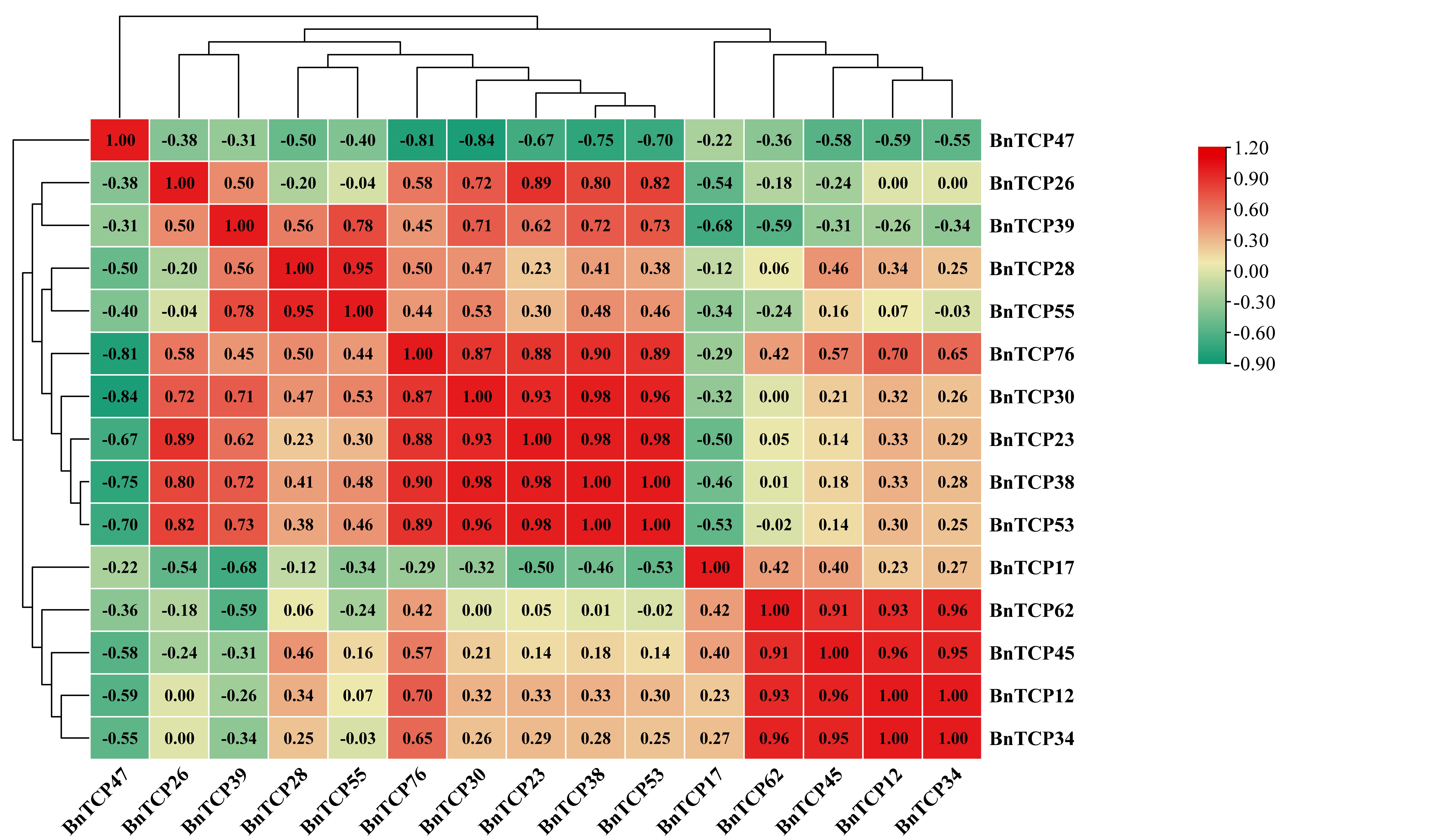

Supplement: Supplementary file 13 — Supplementary file13 (JPG 2112 KB) [file 13205_2025_4273_MOESM13_ESM.jpg]
